# Initial experience with AI Pathway Companion: Evaluation of dashboard-enhanced clinical decision making in prostate cancer screening

**DOI:** 10.1371/journal.pone.0271183

**Published:** 2022-07-20

**Authors:** Maurice Henkel, Tobias Horn, Francois Leboutte, Pawel Trotsenko, Sarah Gina Dugas, Sarah Ursula Sutter, Georg Ficht, Christian Engesser, Marc Matthias, Aurelien Stalder, Jan Ebbing, Philip Cornford, Helge Seifert, Bram Stieltjes, Christian Wetterauer

**Affiliations:** 1 Research & Analytic Services University Hospital Basel, Basel, Switzerland; 2 Institute of Radiology, University Hospital Basel, Basel, Switzerland; 3 University Basel, Basel, Switzerland; 4 Institute of Urology, University Hospital Basel, Basel, Switzerland; 5 Siemens Healthineers GmbH, Erlangen, Germany; 6 Department of Urology, Liverpool University Hospitals NHS Trust, Liverpool, United Kingdom; 7 Danube Private University, Krems, Austria; The University of the West Indies, JAMAICA

## Abstract

**Purpose:**

Rising complexity of patients and the consideration of heterogeneous information from various IT systems challenge the decision-making process of urological oncologists. Siemens AI Pathway Companion is a decision support tool that provides physicians with comprehensive patient information from various systems. In the present study, we examined the impact of providing organized patient information in comprehensive dashboards on information quality, effectiveness, and satisfaction of physicians in the clinical decision-making process.

**Methods:**

Ten urologists in our department performed the entire diagnostic workup to treatment decision for 10 patients in the prostate cancer screening setting. Expenditure of time, information quality, and user satisfaction during the decision-making process with AI Pathway Companion were recorded and compared to the current workflow.

**Results:**

A significant reduction in the physician’s expenditure of time for the decision-making process by -59.9% (p < 0,001) was found using the software. System usage showed a high positive effect on evaluated information quality parameters completeness (Cohen’s d of 2.36), format (6.15), understandability (2.64), as well as user satisfaction (4.94).

**Conclusion:**

The software demonstrated that comprehensive organization of information improves physician’s effectiveness and satisfaction in the clinical decision-making process. Further development is needed to map more complex patient pathways, such as the follow-up treatment of prostate cancer.

## Introduction

In 2017, there were 1.4 million incident cases of prostate cancer worldwide, leading to 416 000 deaths [1]. A combination of increasing incidence rates and an aging population have led to a 42% increase in prostate cancer cases since 2007 [1]. Indeed, the management of prostate cancer patients is a complex process involving an expanding multi-disciplinary team, including urology, radiology, pathology and radiotherapy. Efficient exchange of information among these departments is crucial to deliver appropriate high-quality care and reduce adverse effects. However, in most organizations, relevant data is stored disparate and siloed in various IT systems, and departments operate largely independent of each other [2–4]. These technological gaps between departments prohibit clinicians from building a comprehensive understanding of the patient’s individual condition [5, 6]. In daily clinical practice, clinicians spend a substantial amount of time collecting, integrating, and assessing patient data in order to care for their patients [7].

To provide physicians a holistic view of patients’ medical conditions, a seamless exchange of information across systems and departments is required [8–10]. Effective data integration tools that extract and combine data from multiple sources are supported by integration of best practice guidelines is needed to ensure continuous interdisciplinary patient care [6, 11–13]. Moreover, data representation must be adapted to the individual needs and choices of each recipient and its purpose in the care supply chain to avoid missing or overwhelming information [6].

In the last three years, we have worked together with an industry partner, Siemens Healthineers, on prototypes for pathway-specific clinical decision support systems based upon best practice as defined by the EAU prostate cancer guidelines. This work consisted of both data mapping, data integration and development of front-end data representation. Here, we evaluated the first CE-certified version of this development in the context of prostate cancer treatment decision making.

## Methods

### Guideline statement

All methods were carried out in accordance with relevant guidelines and regulations. The Ethikkommission Nordwest- und Zentralschweiz (EKNZ) provided an exempt from ethical committee approval due to its retrospective design and minimal risk categorization. Patients provided their written informed consent for the collection of their data. Informed consent is collected prior to an examination and is archived as a PDF document in the hospitals clinical information system.

### Clinical decision support software (CDSS)

**Fig 1:**
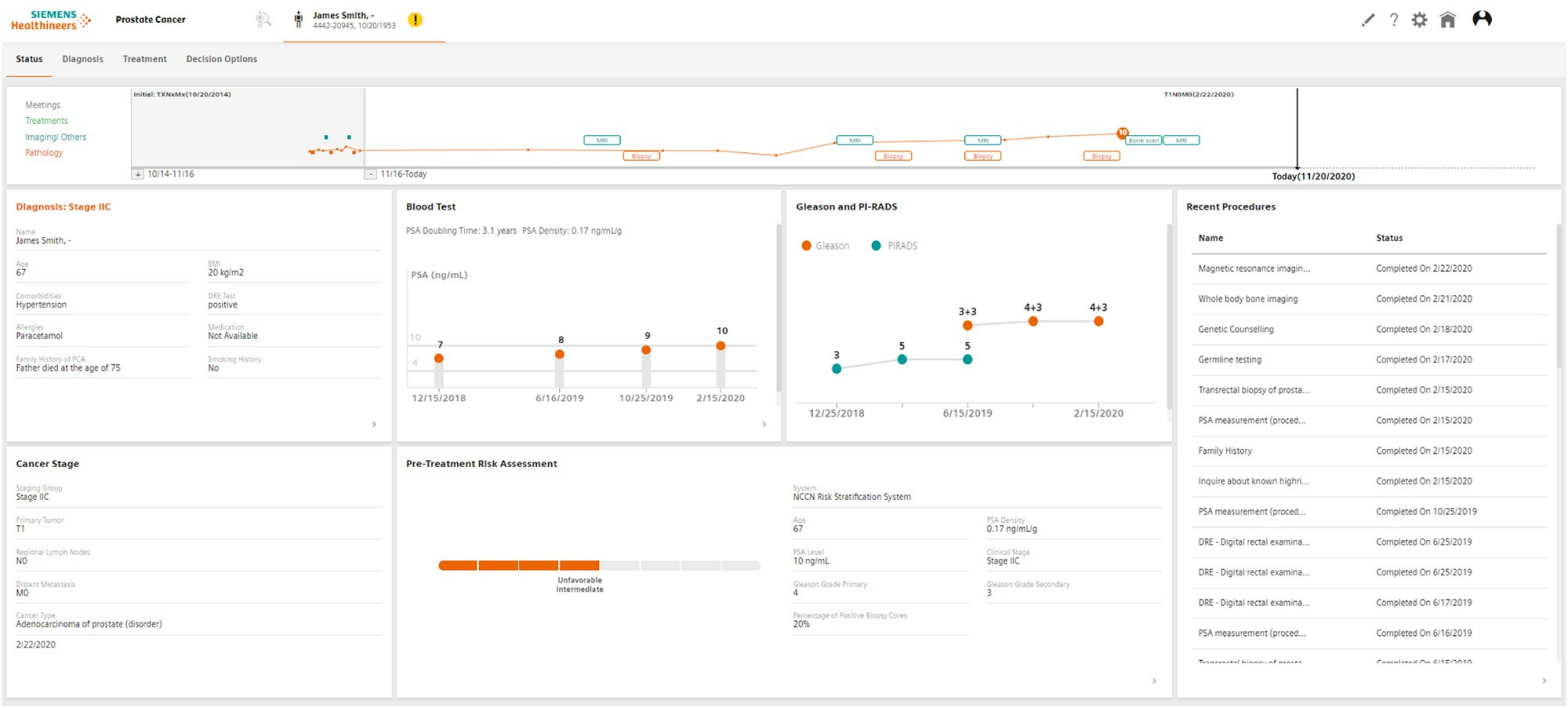
AIPC status page: Aggregates and correlates data from patient longitudinal history, imaging and pathology reports, laboratory results, and clinical studies. Displays a patient’s historical timeline summary of relevant clinical events. Provides historical trends and progression of key biomarkers.

**Fig 2:**
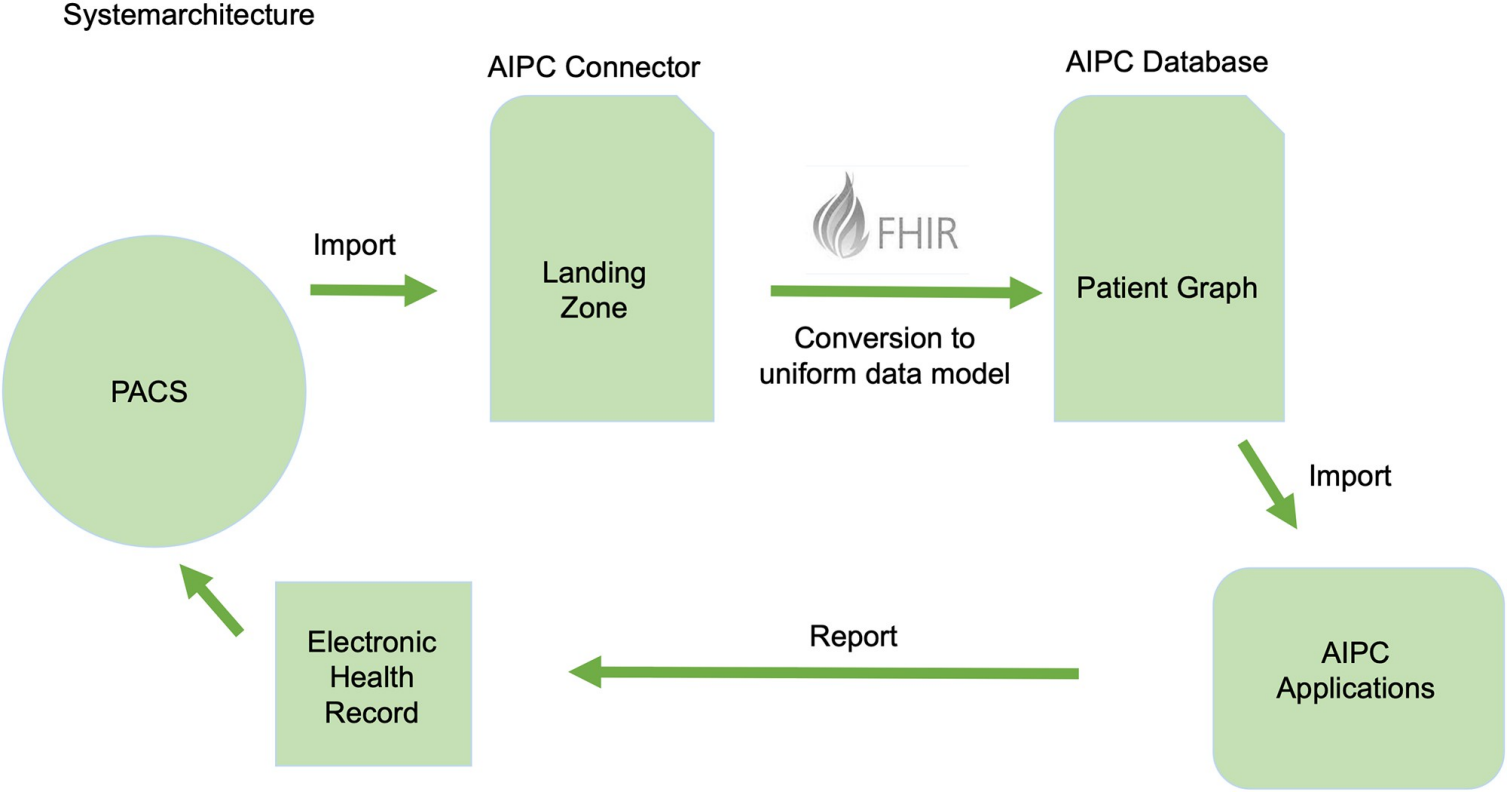
The web-based AIPC product suite consists of several components (AIPC Connector, database and applications). The AIPC Connector is responsible for importing data from various data sources. Imported data (e.g. CSV files, SQL databases, EHR and PACS) are imported from the primary data sources and temporary stored in the Landing Zone. In the Landing Zone data are converted to a uniform data model (FHIR). The AIPC Database can import converted data through an FHIR interface. The data is then made visible to the end user on the AIPC applications in form of Dashboards, MDT reports, radiology images, etc. After decisions for a diagnostic or therapeutic measure have been made, a report can be generated within the AIPC applications for documentation purposes, which can then be sent back to the source system via a shared network file system (electronic health record (EHR).

The clinical decision support software (CDSS) AI-Pathway Companion Prostate Cancer VA10B (Siemens Healthcare GmbH, Erlangen, Germany) was used in this study. It aggregates, correlates, and displays relevant clinical information along the disease-specific pathway in patient centric dashboards, Fig 1. It supports and provides recommendations on diagnostic or therapeutic options on prostate adenocarcinoma cancer by depicting evidence of clinical guidelines in correlation to the patients’ current disease condition. The CDSS Connector system was integrated with the hospital infrastructure to automatically collect all the relevant patient medical information from all the necessary source information systems (e.g., Laboratory Information System, Radiological Information System, PACS, pathology information system). It standardized and structured health data and operational data into a patient data model using standard coding systems (e.g., SNOMED CT, LOINC) and the FHIR standard (Fast Healthcare Interoperability Resources, HL7, hl7.org/fhir). The data structuring included a natural language processing (NLP) unit. An overview of the system’s architecture is provided in Fig 2.

In case of incomplete data extraction by the software (e.g., in case of external paper or scanned documents), missing values were completed manually.

### Study design

We evaluated the utility of the software for routine clinical use and compared it to the traditional routine practice. The diagnostic work-up of all patients included PSA, digital rectal examination, prostate volume, PSA density, highest PIRADS score in prostate MRI (Table 1). In routine practice relevant patient data like clinical examination, MRI, biopsy results and MDT documentation are documented in a traditional narrative report in three different systems (CIS, LIS and RIS). A total of 10 patients undergoing prostate cancer screening were included in the evaluation, which resulted in a total of 200 observations reviewing the same cases in the traditional way and with the software. The time from finding a patient to recommending treatment was measured using the current standard, which includes manual search of information in multiple separated systems, and Siemens AIPC, which provides access to information from all these systems from a single point of entry.

**Table 1:** Case characteristics.

Case Number	Age	DRE	PSA ng/ml	Prostate Volume ml	PSA density ng/ml^2^	Highest PI-RADS	Recommendation
1	73	NS	6.4	28	0.23	4	biopsy
2	72	NS	2.8	27	0.10	3	monitoring
3	58	NS	19.7	48	0.41	5	biopsy
4	71	NS	11.5	64	0.27	5	biopsy
5	57	NS	4.8	37	0.13	4	biopsy
6	71	NS	8.0	56	0.14	5	biopsy
7	68	NS	7.5	19	0.38	3	biopsy
8	62	NS	6.1	52	0.12	4	biopsy
9	65	NS	17.0	109	0.16	5	biopsy
10	64	NS	5.1	36	0.14	5	biopsy

DRE = Digital Rectal Exam, NS = Non Suspicious.

To reduce possible confounds due to case variations, each reader, in a real practice scenario, compiled the same set of screening cases for accessing, integrating, and evaluating patient information once using the traditional method (manual compilation from several systems) and once with the software. All cases were first compiled using the standard approach and afterwards using the software in order to avoid previous opinion bias. In order to further reduce bias, a wash-out period of 4 weeks was instantiated between the sessions. All readers received training on how to use the solution before the actual investigation. Both solutions were applied to the patient’s complete prostate cancer screening pathway from initial screening, diagnostic measures and decision making. For each case, the readers were instructed to perform their respective tasks in the patient management using the two methods (traditional vs. the software). In both scenarios, the goal of a procedure recommendation based upon the underlying information was the same. After all examinations were completed, the results were evaluated by residents and specialists in urology.

### Evaluation

The system’s performance was evaluated based on user satisfaction, data quality, effort for data acquisition, integration, and evaluation. Evaluation of user satisfaction and data quality were based on surveys [14]. The respective questions of all surveys are listed in Tables 2–4. Users were asked to rate the items on a scale of 1–7, with 1 being no agreement and 7 being full agreement. User satisfaction examined the items net benefits of the system, needs for workarounds, and overall satisfaction. Data quality was examined for two dimensions with several items each. Contextual information quality evaluated for completeness, usefulness and relevance of patient information. Representative information quality evaluated for format, consistency, comprehensibility. The effort required for data collection, integration, and analysis was evaluated using the expenditure of time for each task. Data collection included finding the patient’s medical record and retrieving all examination reports. Data analysis included the evaluation of each examination result. Data integration included the overall assessment of the patient’s current status required for treatment recommendation.

**Table 2:** Mean and standard deviation values for total case evaluation times the software vs current method as a function of user.

Task	SPC [sec]	Current method [sec]	Difference [sec]	% Difference	p-Value
Find Patient	6.90	10.40	-3.50	-33.62	< 0.001
Access lab values	4.42	6.65	-2.24	-33.59	< 0.001
Evaluation lab values	3.32	16.52	-13.20	-79.89	< 0.001
Access MRI	17.50	32.92	-15.42	-46.85	< 0.001
Evaluate MRI	5.05	8.99	-3.94	-43.79	< 0.001
Access clinical data	8.15	27.73	-19.58	-70.62	< 0.001
Evaluate clinical data	19.26	66.53	-47.26	-71.04	< 0.001
Data integration	15.01	19.99	-4.97	-24.89	< 0.001
Recommendation	1.83	1.98	-0.15	-7.55	0.051
Total	76.72	191.44	-114.72	-59.93	< 0.001

**Table 3:** Survey results of the effect size for comparison of contextual information quality with current method and the software.

Construct	Item	Cohen’s d	95% CI	
Completeness	“The system provides me with complete information.”	-0.33	-1.28	-1.28
	"The system produces comprehensive information."	2.36	1.13	0.62
Relevance	“Information within the system is applicable.”	0.59	-0.37	1.13
	"Information with the system is relevant for my job."	-0.40	-1.34	3.58
	"In general, information within the system is relevant."	0.16	-0.78	-0.37
Timeliness	The information provided within the system is up-to-date.	0.06	-0.88	1.55
	"The information provided within the system is received in a timely manner."	3.16	1.75	-1.34
Usefulness	“Information within the system is informative.”	1.56	0.49	0.55
	"Information within the system is valuable."	0.88	-0.11	-0.78

**Table 4:** Survey results of the effect size for comparison of representational information quality with current method using traditional reports and the software using charts.

Construct	Item	Cohen’s d	95% CI	
Format	“The information provided within the system is well laid out.”	5.19	3.22	1.10
	"The information provided within the system clearly presented on the screen."	6.15	3.90	-0.88
Consistency	“Information within the system is accurate.”	-0.29	-1.24	1.00
	"In general, information within the system is reliable."	0.00	-0.94	1.75
Understand- ability	“Within the system, information is easy to comprehend.”	2.64	1.36	4.57
	"Within the system, information that is clear in its meaning."	1.50	0.44	0.49

### Statistical analysis

The Wilcoxon signed-rank test was used to compare case preparation time for the traditional method and the software. Cohen’s d was used to compare responses to the survey questions. P < 0.05 was considered statistically significant. Analysis was conducted in R (R Core Team, 2014) and figures were produced using the package ggplot2 [15].

## Results

Characteristics and tasks of the selected cases in Group A are listed in Table 1. Recommendations based on the traditional method and using the software were identical for all observers and all patients. Ten clinicians with an average professional experience of five years (ranging from two to twelve years) participated in the study.

### Expenditure of time

**Fig 3:**
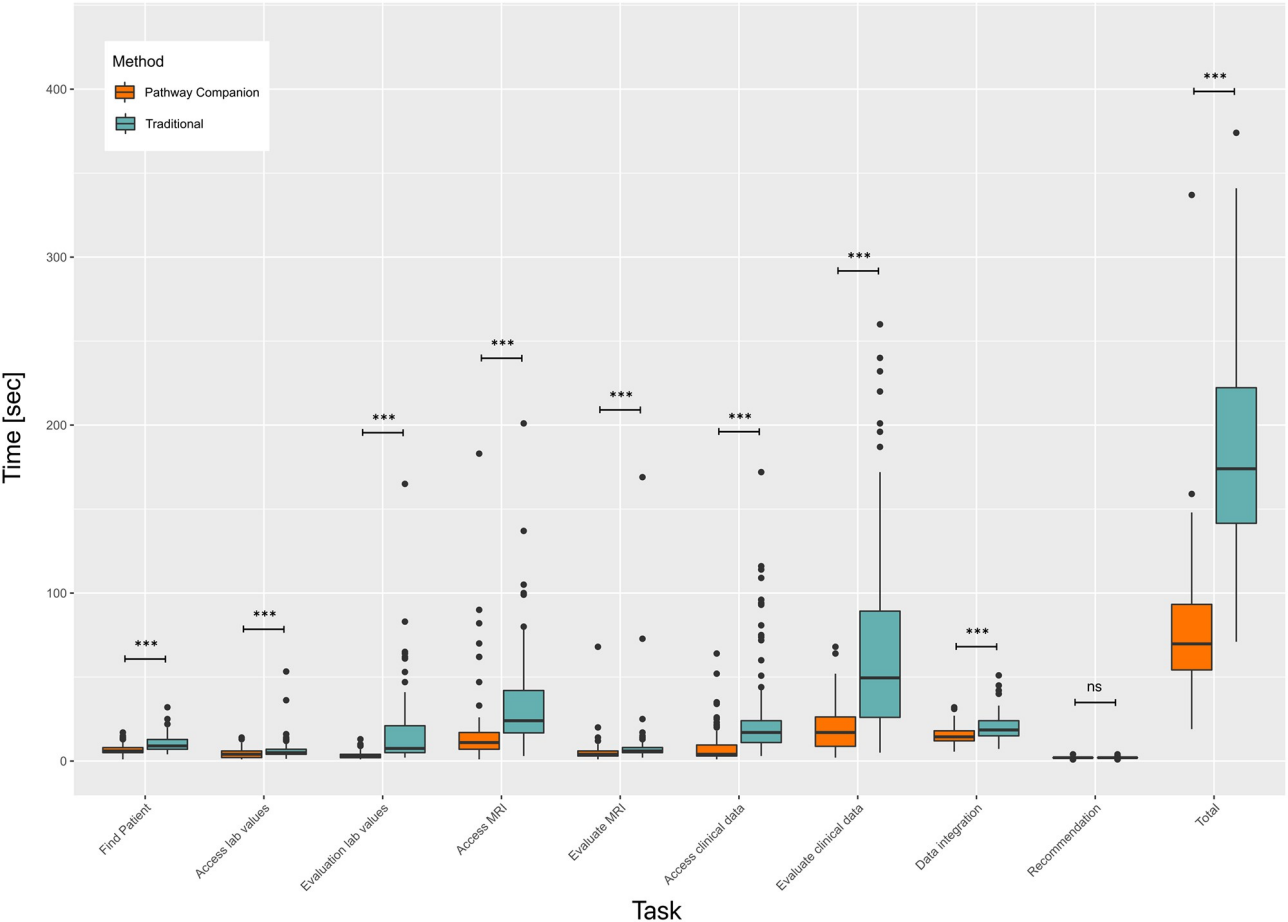
The bar chart shows the time required for a physician to complete the tasks along the prostate cancer screenings pathway. The tasks were completed once with the software and with current method.

In the analysis for paired cases, we compared the times required for the respective tasks, as well as the overall case evaluation time. The total expenditure of time for case preparation was significantly lower using the software as compared to the traditional method (Reduction of 114.7sec; 59.9%; p < 0.001). Detailed results for the respective tasks are displayed in Fig 3 and Table 1.

### Information quality

Contextual and representational information quality was improved in 9 / 15 items examined. The detailed results of the surveys on specific aspects of information quality are shown in Tables 2 and 3. The effect size was calculated using Cohen’s d. Positive values indicate an effect in favor of the software, negative values an effect in favor of the traditional method. Values above 0.2 reflect a low, above 0.5 a medium, and above 0.7 a high effect size.

### User satisfaction

The survey results indicated that the use of the software resulted in overall higher ratings on all of the questions regarding ease of use and satisfaction. Using the software resulted in higher user satisfaction and contributed to a statistically significant improved patient evaluation. No effect on avoidance of the system was observed. Results for overall satisfaction across participants are shown in Table 5. Survey results of the effect size for user satisfaction of current method and the software.

**Table 5:** Survey results of the effect size for user satisfaction of current method and the software.

Construct	Item	Cohen’s d	95% CI	
Net benefits	"Using the ECM system increases my productivity."	4.35	2.63	2.64
Workarounds	"I always look for ways to avoid using the system."	-0.12	-1.06	-0.11
Satisfaction	"I am satisfied with the system."	4.94	3.05	1.86

## Discussion

Healthcare professionals are challenged with increasing complexity of interdisciplinary care and rising amounts of patient data. In order to ensure the quality of care, the integration of data from multiple sources is needed [7]. In this study, we present a new software to provide comprehensive patient information and support physicians in evaluation of patients’ health conditions. Using prostate cancer screening as an example, the software reduced the time needed to evaluate the patient’s health condition compared to the previous method. Contextual and representative data quality was improved in 9/15 criteria examined. Users showed higher satisfaction using the new software.

Our results further substantiate that of previous studies, which assessed the value of clinical decision support systems for several medical conditions and demonstrated a reduced case evaluation time by using patient-centered organization and representation of information [16, 17]. The software reduced the manual effort for collecting and preparing patient information. The task analysis further emphasizes the impact of this manual effort in examination results with a large amount of data, storage of information in independent systems and poorly structured reports. The use of the new software reduced the evaluation of laboratory values as a report with high information density by 80% through the targeted preparation of task specific values. The access to radiological examination results, which are normally stored in a separate information system, was reduced by 47%. The evaluation of highly unstructured clinical information could be reduced by 71% due to structured representation.

The survey results on contextual information quality showed a high positive impact of the software on the completeness and timeliness of the information. Previous studies have shown the importance of completeness of information to reduce the failure of discussion rates [18]. Reducing failure to discuss rates can shorten time to treatment initiation and thus improve patient outcomes. Moreover, the dashboard-based representation showed high impact on format and understandability of data, which is required for a comprehensive overview of patient health status. This is in line with previous studies which showed that clinical decision support systems have the potential to support the use of the most relevant information and avoid treatment errors [19]. The use of the software showed a high positive impact on user satisfaction. The software enables to focus on patient counseling and shared decision making instead of being stuck in poorly organized electronic medical records.

This study is limited by being confined to a single institution. Thus, it is difficult to generalize our results to other institutions using other IT systems with different data structure and data quality. Due to the fact that only 10 readers were observed, the ability to assess inter-user variability was limited. Nevertheless, the results based on 10 patient cases led to statistically significant observations.

A further limitation is the fact that we only assessed the linear pathway of pretherapeutic prostate cancer management, however our findings are consistent with previous studies on clinical decision support system in different settings [14, 16, 20–23]. As our study only serves as an assessment of pre-therapeutic prostate cancer management, further studies are required to evaluate the usability of the software in the setting of post-therapeutic prostate cancer management as well as in advanced cancer stages. The benefits of clinical decision support systems may be even higher with more complex cases. Nevertheless, this study clearly demonstrates the potential of the software to reduce consultation preparation time in prostate cancer patient care. In the future, the use of automated risk calculators has the potential for further time saving. These results are very important, as with an increasing emphasis on value-based healthcare, quality time between physician and patient has become a precious resource. All practicing physicians wished they had more time for a given patient and will thus embrace software solutions that save time for more meaningful engagement with patients.

## Conclusion

Using the software significantly reduced consultation preparation times in pre-therapeutic prostate cancer management as compared to the classic solution. The software effectively improved the decision-making process and user satisfaction. Further work is needed to investigate the effect in post-therapeutic prostate cancer management.

## Abbreviations

AI: Artificial Intelligence
AIPC: Artificial Intelligence Pathway Companion
CIS: Clinical information system
CT: Computed tomography
DICOM: Digital Imaging and Communications in Medicine
EHR: Electronic health record
IPSS: International Prognostic Scoring System
LIS: Laboratory information system
MRI: Magnetic resonance imaging
PIRADS: Prostate Imaging Reporting and Data System
PSA: Prostate-specific antigen
RIS: Radiology information system
